# Brain Tumors in NF1 Children: Influence on Neurocognitive and Behavioral Outcome

**DOI:** 10.3390/cancers11111772

**Published:** 2019-11-11

**Authors:** Matilde Taddei, Alessandra Erbetta, Silvia Esposito, Veronica Saletti, Sara Bulgheroni, Daria Riva

**Affiliations:** 1 Developmental Neurology Unit, Fondazione IRCCS Istituto Neurologico Carlo Besta, Via Celoria 11, 20133 Milan, Italy; matilde.taddei@istituto-besta.it (M.T.); silvia.esposito@istituto-besta.it (S.E.); veronica.saletti@istituto-besta.it (V.S.); daria.riva@istituto-besta.it (D.R.); 2 Neuroradiology Unit, Fondazione IRCCS Istituto Neurologico Carlo Besta, Via Celoria 11, 20133 Milan, Italy; alessandra.erbetta@istituto-besta.it

**Keywords:** neurofibromatosis type-1, children, optic pathways glioma, brain tumor, cognitive-behavioral outcome

## Abstract

Neurofibromatosis type-1 (NF1) is a monogenic tumor-predisposition syndrome creating a wide variety of cognitive and behavioral abnormalities, such as decrease in cognitive functioning, deficits in visuospatial processing, attention, and social functioning. NF1 patients are at risk to develop neurofibromas and other tumors, such as optic pathway gliomas and other tumors of the central nervous system. Few studies have investigated the impact of an additional diagnosis of brain tumor on the cognitive outcome of children with NF1, showing unclear results and without controlling by the effect of surgery, radio- or chemotherapy. In the present mono-institutional study, we compared the behavioral and cognitive outcomes of 26 children with neurofibromatosis alone (NF1) with two age-matched groups of 26 children diagnosed with NF1 and untreated optic pathway glioma (NF1 + OPG) and 19 children with NF1 and untreated other central nervous system tumors (NF1 + CT). NF1 + CT and NF1 + OPG showed significantly impaired cognitive abilities compared to NF1 group, with weaknesses in visuo-spatial abilities, visual scanning and verbal working memory, while general verbal abilities are preserved. Moreover, NF1 + OPG patients present more frequent internalizing problems and increased oppositional-deviant behaviors. These results suggest that the co-diagnosis of a brain tumor in NF1 children may partially worsen the cognitive and emotional outcome.

## 1. Introduction

Neurofibromatosis type-1 (NF1) is an autosomal dominant genetic disorder with an incidence of approximately 1 in 2700 individuals [1]. Individuals with NF1, in addition to clinical characteristics (café-au-lait spots, multiple neurofibromas, and bone deformities), also have a high incidence of macrocephaly, T2-weighted hyperintensities (T2H) with no mass effect and no contrast enhancement (sometimes referred to as unidentified bright objects-UBOs) and are particularly prone to develop tumors of the central nervous system [2,3]. In adults, high-grade gliomas may occur, whereas in children, the most commonly-encountered brain tumor is a low-grade glioma, a World Health Organization grade I tumor (pilocytic astrocytoma) with low mitotic rates and low proliferative indices [3,4]. While these pilocytic astrocytomas can occur anywhere in the brain, they are most frequently detected in the optic pathway and brainstem. The optic pathway gliomas (OPGs) affect 15–20% of NF1 patients with a mean age between 3 and 6 years of age [5,6]. Another 3% to 5% of patients have other types of brain tumors [7], mainly located in the brainstem and the cerebellum, but they can also be supratentorial [2,7].

The behavior of NF1-OPGs can be unpredictable, requiring that all children with NF1 undergo routine surveillance [3]. Initial management of optic pathway gliomas is usually observation with serial and frequent magnetic resonance imaging (MRI) scans and ophthalmologic examinations. Most tumors remain indolent, and when they progress, it is often slow in pace [8]. Once an OPG has been identified, the frequency of neuroimaging and visual assessment depends on the site of the tumor, degree of visual impairment and associated symptoms, as well as evidence of progressive disease [5]. There is no consensus on the specific interval of neuroimaging and visual assessments, but most centers experienced in treating patients with NF1-associated OPG perform eye examinations and vision testing every 3 months for the first year after diagnosis, with increasing intervals thereafter [3]. Treatment is reserved for patients with a documented decline in visual acuity or significant tumor progression on MRI scan with associated symptoms and signs [8].

Although not included as NF1 diagnostic criteria, studies have reported that over 60% of children with NF1 also have learning, attention and behavioral delays [9,10,11]. In particular, visual-spatial impairment has long been considered a hallmark feature of the disorder [12], while evidence pertaining to other areas of cognition, such as executive and motor functioning, verbal memory, and various linguistic skills, remains inconsistent [10].

Moreover, affective and behavioral dysregulation have been extensively described, such as attention problems, problems reflecting anxiety, depression, or withdrawal and social problems [13]. These cognitive-behavioural impairments have a substantial impact on the quality of life and are a major concern for parents and teachers [14].

In literature, some attempts have been made to correlate these cognitive and neurological deficits with radiological findings. Most of the studies addressed the correlation with T2H, revealing inconsistent results [15,16,17,18,19]. The relationship between T2H and neuropsychological problems has been studied in different ways by considering, for example, the relation between cognitive impairments and the presence or absence of T2H, their number, size, and specific locations. Although the results of these studies are still heterogeneous, some studies have found that the localization of T2H in the thalamus is related to a lower Intelligence Quotient (IQ) [19,20].

Some studies on optic pathways and extra-optic pathways low grade gliomas investigated the influence of surgery or pharmacological treatment on neurocognitive functioning [21,22,23]; however, only 30–50% of low grade tumors are symptomatic in NF1, and only one-third of affected children will require therapeutic intervention [24,25]. Studies examining neuropsychological status of NF1 children with non-treated brain tumors are, in our knowledge, only two. Moore and colleagues [26] evaluated the effects of NF1 with and without a codiagnosis of a brain tumor. De Winter et al. [27] replicated this result in a larger group of children with NF1, 24% of whom had also been diagnosed with a brain tumor; in both studies, although no statistically significant differences were found between the groups, there was a consistent trend for those with NF1 plus a brain tumor to score slightly lower than those with NF1 alone.

For what concerns emotional and psychosocial functioning, a recent review investigated psychosocial features of NF1 in children and adolescents [13]. Compared to unaffected children and adolescents of the general population, pediatric patients with NF1 have an increased risk of having social difficulties, mental health disorders, behavioral and emotional problems, as well as diminished quality of life. Studies investigating the disease-related conditions that may worsen the psychosocial outcomes, mostly considered the impact of NF1-related physical symptoms, such as scoliosis [28], optic pathway gliomas [29], and visible or disfiguring manifestations of the disease [30], such as neurofibromas, that can limit physical functioning and impact psychological well-being [31,32].

In our knowledge, no studies investigated the impact of central nervous system tumors on behavioral, cognitive and emotional features of NF1 children who never received any kind of treatment.

In the present monoinstitutional study, we examined the influence of brain tumors on the cognitive and behavioral outcome of 71 children with NF1, comparing children with neurofibromatosis alone with two age-matched groups of children diagnosed with neurofibromatosis and untreated optic pathway glioma and children with neurofibromatosis and non-treated other central nervous system (CNS) tumors.

We hypothesized that the children with NF1 plus a brain tumor would present more pronounced cognitive and emotional-behavior impairment compared to children with NF1 without an additional diagnosis of brain tumor; we hypothesized that tumors involving different central nervous system structures (i.e., OPG vs. other CNS tumors) may differently influence cognitive and emotional-behavioral outcome.

## 2. Results

### 2.1. Intelligence and Psychomotor Development

Table 1 shows Mean, Standard Deviation and group comparison on the Intelligence and Developmental IQs.

One-way ANOVA and post-hoc tests corrected for multiple comparisons (Bonferroni) showed that children with NF1 + OPG and NF1 + CT performed significantly lower than NF1 at mean Full Scale IQ/General Quotient (GQ) and Performance sub-quotients, though still within the normal range. No significant differences among groups has been found for verbal IQ.

In accordance to these results, frequencies analyses showed a higher prevalence of intellectual deficit among groups NF1 with tumors compared to NF1 without tumors (χ2 = 10.863, *p* = 0.004).

ANOVA including as dependent variables the Wechsler scales sub-tests scores, showed significant group differences in the Block Design test, Digit Span test, Symbol Search test; post-hoc tests for Block Design confirmed weaker performances in both groups of NF1 with tumors compared to NF1 without tumors, while for the other sub-tests Bonferroni post-hoc revealed significantly weaker performances for the NF1 + CT group compared to NF1 (Table 1). T-test for independent samples comparing the two groups of patients with tumors taken together to NF1 non-tumor group, confirmed all the significant differences revealed by one-way ANOVA except for Digit Span test.

NF1 + OPG with the involvement of posterior hypothalamic structures present less efficient cognitive abilities than those without hypothalamic involvement (Mean (M) = 84.50; Standard Deviation (SD) = 21.99 vs. M = 87.71; SD = 18.13), though this difference is not significant. For what concerns NF1 + CT, any relevant differences have been found comparing children with tumor in the brainstem or in the hypothalamus vs. tumors in other areas.

### 2.2. Psychopathological Outcome

By one-way ANOVA the NF1 + OPG showed significant higher levels of oppositional-deviant behaviors compared to the non-lesion NF1 group and to NF1 + CT, with significant differences between NF1 + OPG and NF1 + CT at the post-hoc tests.

The χ2 showed significant differences among the three groups according to Child Behavior Checklist (CBCL) Total Internalizing scale; in particular, the NF1 + OPG group showed a higher number of subjects with scores in pathological ranges (Adjusted Pearson Residuals = 1.6), while in the NF1 group the presence of borderline scores is significantly more frequent (Adjusted Pearson Residuals = 2.9). No significant results have been found for CBCL Externalizing and Total Problems scores (Table 2).

No other differences at CBCL scores have been found in accordance to the location of the tumor.

### 2.3. Influence of IQ on Psychopathological Outcome

In the whole sample, without considering the presence of brain tumors, the total IQ is negatively correlated to CBCL Total Externalizing (*p* = 0.005; Pearson R = −0.341) and Attention Problems (*p* < 0.001; Pearson R = −0.458) scores, also surviving the correction for multiple comparison, revealing that lower IQ is associated with higher level of behavioral problems. Moreover, IQ revealed a significant covariate of three group factor (NF1, NF1 + GVO, NF1 + CT) for Oppositional-deviant Problems, Attention Problems and CBCL Total Externalizing scores (Table 3).

### 2.4. Role of T2H and Peripheral/spinal Neurophibromas

The information about the number and location of T2H is available for 69 patients. No significant results have been found in the two groups with tumor according to T2H Nr or localization.

The presence of T2H in the Thalamus is related to higher levels of withdrawn behaviors (T-test p=0.008) and affective problems (T-test *p* = 0.017) in NF1 subjects without tumors. The information about additional presence of spinal and/or peripheral neurofibromas is available for 67 patients. Additional neurofibromas are significantly more frequent in the two group of lesion (NF1 = 4%, 1/24; NF1 + OPG = 39%, 9/23; NF1 + CT = 26%, 5/19) and are related to higher frequencies of borderline or pathological scores at Anxiety-depression (*p* = 0.027; χ2 = 4.875) and Somatic lamentation (*p* = 0.027; χ2 = 4.875) scales in the whole sample.

The presence of peripheral and/or spinal neurofibromas is equally distributed in the two sample of children with tumor. Patients with spinal and/or peripheral neurofibromas are older than those with brain tumors without neurofibromas (NF+ = 139.14 ± 49.23; NF− = 80.70 ± 41.84; *p* < 0.001) and have less number of T2H (NF+ = 3.43 ± 1.65; NF− = 5.46 ± 1.53; *p* < 0.001), in accordance to the standard disease course. 

Moreover, considering the two samples with tumors, the presence of neurofibromas is not associated to decreased cognitive abilities, while a higher levels of somatic lamentations and anxiety symptoms (T-Test for independent samples *p* = 0.015 and *p* = 0.010) and more frequent borderline levels at CBCL Total Internalizing problems (*p* = 0.045; χ2 = 6.181) have been found in children with brain tumor and additional peripheral neurofibromas.

## 3. Discussion

The present study adds further insight on cognitive and emotional-behavioral characterization of NF1 children with central nervous system tumoral lesions (optic pathway glioma and non-optic pathways tumors). In particular, we evidenced that children with NF1 and the additional diagnosis of tumors showed decreased intellectual abilities compared to NF1 without associated tumors, with cognitive weaknesses in visuo-spatial abilities, visual attention and verbal working memory, while general verbal abilities are preserved.

We also found that NF1 patients with brain tumors have higher levels of emotional and behavioral problems, involving both externalizing and internalizing domains, despite the average level of behavioral difficulties is not pathological. In particular, the NF1 + OPG have higher levels of internalizing and oppositional behaviors compared to NF1 + CT.

The results on cognitive outcome in NF1 children with and without brain tumor confirm and give statistical strengths to the earlier findings of De Winter [27] that the cognitive and visuo-spatial deficits in children with NF1 are impacted by the additional diagnosis of brain tumor.

In our sample, any significant correlation emerged between decreased cognitive abilities and neuroradiological features, such as tumor localization (optical pathways with or without the involvement of hypothalamic structures, brainstem or supratentorial areas), T2H number or localization.

In our sample, most of the non-optic tumors were localized in the brainstem; the six subjects with tumor localized in supratentorial regions (one in the thalamus, three in the hypothalamus, one in the basal ganglia and one in the third ventricle) showed cognitive weakness in digit span test, without statistically significant impairment.

This lack of statistical significance may be due to the small sample size and the reduced neurological variability for what concerns the two subsamples with tumors, or rather the cognitive deficit is likely not related to the direct influence of the lesion, but could be the phenotypic expression of a general clinical disease severity correlated to co-etiological genetic factors [6].

For what concerns behavioral and emotional assessment, the CBCL findings evidenced that children and adolescents with NF1 and additional diagnosis of brain tumor more often present pathological internalizing traits (i.e., introversion, social withdrawn, anxiety symptoms), together with higher levels of oppositional-deviant behavior. These results are in line with previous study in literature about social, emotional and behavioral outcomes in children with brain tumor in general [23,33]; moreover, it seems that the social and emotional vulnerability showed by children with NF1, despite highly variable [34], may be worsened by the presence of central nervous system involvement.

For what concerns CBCL results, it is hard to propose an interpretation based on a functional-structural correlation; in fact tumors were mainly localized in the optical pathways and the brainstem, as usually reported in NF1 [2], structures not involved in higher level neuropsychological functions and emotional processing. Thus, the higher prevalence of psychopathological traits could be either related to an effect of the genetic background on brain structure or function, as discussed for cognitive abilities, or may be a secondary result of specific complications of the tumor and of the burden to cope with particularly severe illness [31,34].

In our sample, pathological levels of internalizing traits seem to be more frequent in the NF1 + OPG sample than in patients with extra-OP tumors. Optic-pathways gliomas and brainstem tumors in NF1 children are usually less aggressive than their counterpart in non-NF1 children and, from an oncologic point of view, show a good prognosis [35,36,37]. However, at long-term follow-up, also children with low-grade tumors may display impairments, disabilities, handicaps, and a low quality of life, depending on tumor site, age, and disease recurrence [33]. In addition, children with optic glioma, also when asymptomatic, are subjected to frequent (every three months after the discovery of the tumor) and stringent medical controls, as detailed examination should include age-appropriate measures of visual acuity, visual field assessments, funduscopic inspections, MRI and visual evoked potentials. This follow-up is mandatory and drives the decision to start treatments in case of enlargement of the tumors and/or worsening of visual functions. This could increase the level of stress experienced by the children and explain the higher presence of internalizing and externalizing problems.

With the aim to better characterize the two samples with brain lesions, we evaluated additional neurological features that may impact on the cognitive and emotional-behavioral outcome in NF1 children, such as T2H numbers / location, and presence of peripheral and/or spinal neurofibromas.

In particular, peripheral and/or spinal neurofibromas, are medical conditions related to higher severity of the disease and have been correlated to high frequency and severity [38,39] of pain, which can negatively impact quality of life [30,40] and thus influence the patients’ psychological well-being.

Among the two groups of lesions, the presence of peripheral/spinal neurofibromas is associated to higher internalizing problems; despite the two groups of tumor did not differ significantly in accordance to this variable, we found a higher frequency of children with additional peripheral and/or spinal tumor among NF1 + OPG group (39.1% vs. 26.3%), that may partially explain the higher level of behavioral problems in this group.

The number and localization of T2H did not reveal a predictive variable of cognitive-behavioral outcome in the NF1 children with tumors; however, in NF1 without tumors higher levels of anxiety and withdrawn problems are related to the presence of T2H in the thalami, relevant integrative structures with high number of connections to other cortical and sub-cortical areas implicated in emotion and cognition [41].Our results are in line with previous findings about reduced influence of T2-weighted hyperintensities on cognitive profile in NF1, in terms of total number, size, and location in the whole brain and in the basal ganglia, cerebellum, brain stem, and thalamus [17,18,42]. Many possible causes can be advocated to explain these results, first of all related to the modifiable and transitional nature of these neuroradiological feature [11,42,43]. Moreover, the presence of T2H in the same locations in almost all patients limited the assessment of potential impact on cognitive-emotional functioning. The role of T2H in influencing emotion and cognition in NF1 is still under debate and further data are necessary.

Another interesting result concerns the role of cognitive abilities in predicting behavioral problems: the presence of decreased intellectual abilities is associated to high level of externalizing behaviors; moreover, IQ revealed a significant factor influencing the extent of externalizing problems in addition to the presence of brain tumors. These data confirms that cognitive skills can be a facilitator of functional adaptation to the context and behavioral functioning in children with NF1 [44,45,46].

Despite the fact the IQ distribution in our sample is highly variable, with a percentage of patients with cognitive delay (QI <65) of 19.7%, more frequent than what reported in larger studies on NF1 populations (4% to 8% [11]), the total sample IQ is in the average range (90.43, DS = 18.23) and is in line with what previously reported for NF1 children [15,16,45,47]. The mean IQ in our group of NF1 without tumors, though perfectly in line with what reported in other studies investigating the effects of central nervous system involvement on NF1 cognition, is few point higher than what reported in some studies for NF1 subjects in general and could lead to suspect for the presence of biased group selection on IQ.

The debate on IQ levels in NF1 children presents limitations difficult to overcome, regarding the lack of coherence in terms of IQ instruments and definition among different studies. Most of the studies about NF1 neuropsychological characterization excludes patients with tumors or with other central nervous system involvement, considering it as a confounding factor. On the contrary, the present study aimed at verify whether the down-shifting IQ described in NF1 population could be more pronounced in those with additional central nervous system involvement. Thus, the presence of central nervous system tumor and the tumor localization were the only independent variables; we a-priori excluded sex and age as confounding factors and tested the IQ as covariate on the behavioral and emotional functioning, with the aim to exclude a confounding effect of intellectual disability on the CBCL results.

Studies investigating the effects of central nervous system alterations on NF1 cognitive outcome, showed mean IQ in NF1 without central nervous system involvement (e.g., tumors or T2Hs) quite similar to ours or a few point decreased but with higher SD [16,18,27]. However, with the aim to control for possible selection bias on IQ, we repeated the analyses excluding three subjects with moderate-severe cognitive delay and IQ differences between groups are still significant.

This study has strengths and limitations. Strengths are represented by the subjects’ recruiting among patients afferent to same Institute specialized in neurofibromatosis, which ensures methodological homogeneity in neurological and neuropsychological assessment, in radiological evaluation and in the follow-up protocols. Another positive point is that, so far, this is the first study addressing cognitive and behavioral evaluation comparing the patients in three groups well selected and before any kind of treatment.

The main limitation is the small sample size, still in line with other studies, mainly due to the difficulty in recruiting children with NF1 and un-treated extra-optical tumors. Moreover, we did not investigate the influence of hormonal functioning, that is frequently altered in NF1 patients and can worsen/unmask neurocognitive functions [48]. In particular, deficiency in the growth hormone-insulin-like growth factor has been reported to influence Full scale IQ, Comprehension, Processing speed and Motor abilities in children [49]. This is particularly true for patients with tumor involving the optic chiasm, pituitary gland, and hypothalamus [50,51], who frequently present symptoms of hypothalamic-pituitary function’s disruption such as obesity, behavioral chances, sleep problems and hormonal dysfunctions. Despite our patients are screened for hypothalamic-pituitary functions, these data could not be collected from the endocrinologist, thus the influence of hormonal alterations could not be assessed.

Finally, the psychometric instruments employed for the cognitive assessment are heterogeneous, limitation that is only partially overcome by using the standard scores. For the assessment of the emotional functioning, the current study used data provided only by parents; in future studies the role of parental stress and other environmental factors should be addressed, as variables that could influence parental rating and impact on pathological behaviors. The use of CBCL for pre-school age children, less sensitive to pathological cut-off than the school-age version, could have partially underestimate the differences between groups. Future studies with increased sample sizes could provide homogeneous sub-samples of school-age vs. pre-school age children to be compared.

## 4. Materials and Methods

### 4.1. Participants

Participants of this case-control study were recruited from patients attending a clinical setting specialized in the care of children with NF1 of the Foundation Istituto di Ricovero e Cura a Carattere Scientifico (IRCCS) Neurological Institute Carlo Besta in Milan, Italy, from 1988 until June 2019. Among 198 patients with NF1 that performed neuropsychological assessment, we first selected patients with NF1 and additional central nervous system tumors which did not underwent to any surgical removal and/or pharmacological treatment and/or radiotherapy at the time of cognitive and behavioral evaluation or before (NF1 + CT). Subsequently, we selected a group of patients with NF1 and additional non treated optical pathway glioma (NF1 + OPG) and a group of patients with NF1 alone (NF1) matched by age and sex. Matching was done blindly, without regard to the patient’s identity or test scores. The final groups were composed by 26 NF1, 14 males and 12 females aged between 22.5–195.0 months; 26 NF1 + OPG, 11 males and 15 females aged between 30.5–206.0 months; and 19 NF1 + CT, 13 males and 6 females aged between 26.5–185.0 months. The three groups’ distribution for sex and age are presented in Table 4. 

**Figure 1:**
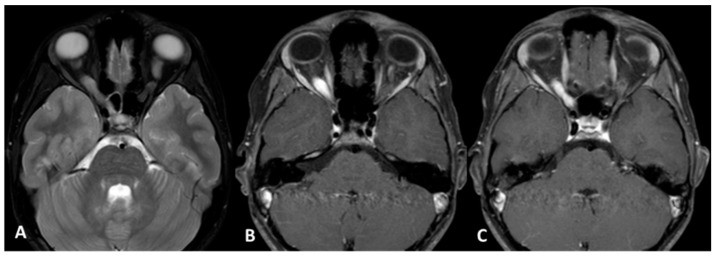
Optic glioma 1Aright (Subject 12 OPG). Orbit MRI. (**A**) Axial T2-weighted images (w.i.) with fat suppression; (**B**,**C**) Axial T1 w.i. with fat suppression and after gadolinium administration show enlargement of the optic nerve in the orbit and in the optic canal on the right side with intense and homogenous enhancement. OPG: Optical pathways glioma; MRI: Magnetic resonance imaging.

**Figure 2:**
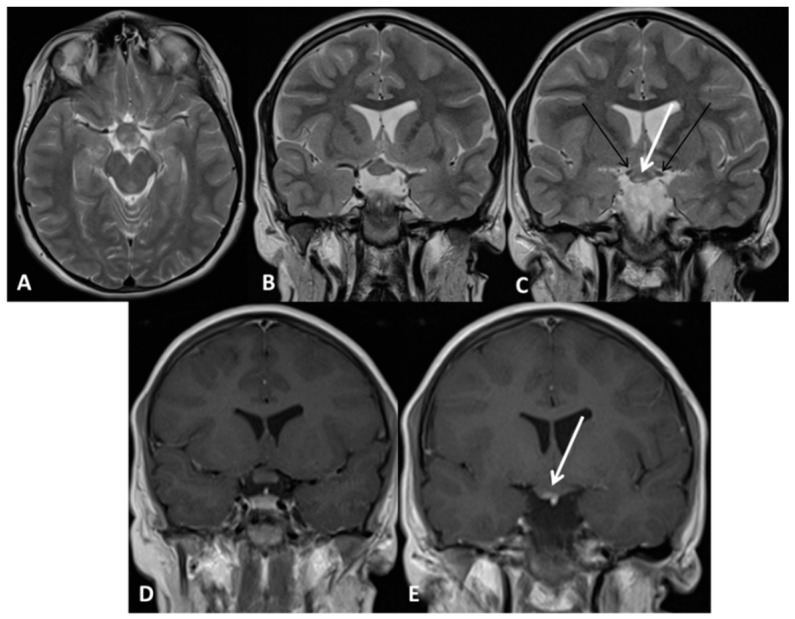
Chiasm and hypothalamic glioma 2aH+ (Subject 09 OPG). Brain MRI. (**A**) Axial T2 weighted images (w.i); (**B**,**C**) Coronal T2 w.i. (**B**,**C**); Panel A, B and C show enlargement of the chiasm and hypothalamus with a small hyperintense area (white arrow in C), while retrochiasmatic tracts are spared (black arrows in C). (**D**,**E**) Coronal T1 w.i. after gadolinium administration showing enhancement in the hyperintense corresponding area (arrow in E). OPG: Optical pathways glioma; MRI: Magnetic resonance imaging.

**Figure 3:**
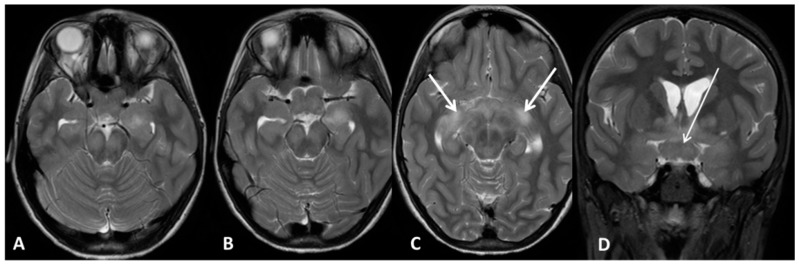
Optic pathways glioma 3B H+ (Subject 16 OPG). Brain MRI. (**A**,**B**,**C**) Axial T2 weighted images (w.i.); (**D**) coronal T2 w.i. with fat-suppression. All the panels show enlargement of the chiasm (arrow in D) and retrochiasmatic visual pathways with abnormal signal intensity (arrows in C). OPG: Optical pathways glioma; MRI: Magnetic resonance imaging.

**Figure 4:**
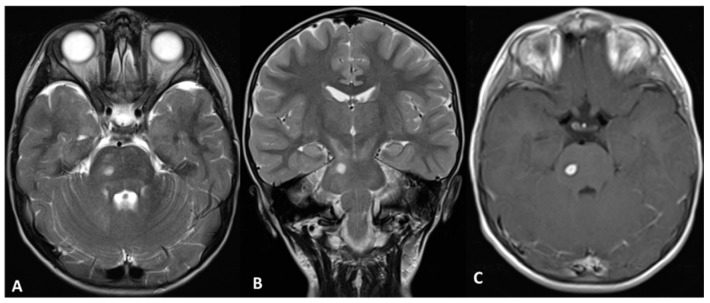
Brainstem Glioma (Subject 05 CT). (**A**,**B**) Axial and Coronal T2 weighted images (w.i.); (**C**) axial T1 w.i. after gadolinium administration show diffuse abnormal signal intensity in the pons and a spot of circumscribed hyperintensity with enhancement consistent with a low-grade glioma. CT: Central nervous system tumor.

Clinical evaluation included neurological examination, MRI, genetic testing, ophthalmological examination including assessment of vision, refraction, biomicroscopy and fundoscopy, and dermatologic evaluation. NF1-OPG children underwent also visual evoked potentials to complete the visual assessment. Additionally, children who presented tumors with hypothalamic involvement underwent to hormonal screening. For patients with tumors, the presence of optic pathways glioma (optic nerve, optic chiasm, with or without involvement of the hypothalamic structures) or other lesions of the central nervous system was assessed by expert neuroradiologist (A.E.) and information about the site of the lesion are available for all the NF1 + CT subjects and 24 out of 26 NF1 + OPG subjects (Table 5 and Table 6). All patients had low grade lesions at the time of cognitive evaluation. Figure 1, Figure 2, Figure 3 and Figure 4 show different brain lesions.

In all cases diagnosis was based on radiological criteria. In the case of asymptomatic tumors, the diagnosis was considered in presence of two or more of the following radiological features: expansive lesion, contrast enhancement or mass effect. The differential diagnosis was considered in non-expansive T2-weighted MRI lesions without contrast enhancement and mass effect [53,54]. For cases who presented tumoral lesions in more than one area the categorization of the subject as OPG vs. CT has been done in accordance to the most affected region.

All the subjects’ parents gave their informed consent for personal data use for scientific aims before they participated in the study. The study was conducted in accordance with the Declaration of Helsinki. The formal approval from the ethic committee and ethical code assignment are not required in accordance to current regulation.

### 4.2. Intelligence and Psychomotor Development

All subjects were assessed in a quiet room by examiners who had specific training on the child assessment. For 54 school-age children, Intelligence was evaluated using the Wechsler Intelligence Scale for Children-Revised (WISC-R) third (WISC-III) or fourth (WISC-IV) editions [55,56,57]. Six pre-school age children (one NF1, two NF1 + OPG and three NF1 + CT) were assessed by Wechsler Pre-school and Primary Scale of Intelligence-III [58]; one pre-school NF1 + OPG girl was assessed by Wechsler Pre-school and Primary Scale of Intelligence-Revised; thus, a total of 61 participants (NF1 *n* = 24, NF1 + OPG *n* = 22, NF1 + CT *n* = 15) performed Wechsler scales and Full Scale IQ, Verbal IQ and Non-verbal Quotient were calculated.

Different versions of the Wechsler scales are composed of different subtests. In general, the verbal domain includes the assessment of verbal fluency and word knowledge (Vocabulary), concept formation and verbal abstract reasoning (Similarities), social knowledge, practical judgment in social situations (Comprehension), general cultural knowledge (Information), short-term verbal memory and attention (Digit-span), verbal sequencing abilities and verbal working memory (Letter-Number sequencing), mental arithmetic ability (Arithmetic). Non-verbal domain assesses visual-spatial visualization and analysis and nonverbal concept formation (Block design), perceptual reasoning (Matrix Reasoning) and categorical reasoning (Picture Concepts), logical and sequential story organization (Picture arrangement), visual-motor integration speed (Coding), visual scanning and selective attention speed (Symbol search). The complete Intelligence scale sub-tests profile is available for all the subjects except for one NF1 + OPG patient which performed the intelligence evaluation elsewhere.

The Wechsler scales’ raw scores were converted to age-corrected sub-tests standard scores normally distributed with a mean of 10 and SD of 3. The sum of age-scaled scores was converted to an overall standard score, with a mean of 100 and standard deviation of 15.

For ten pre-school age children (two NF1, four NF1 + OPG and four NF1 + CT), Griffiths Mental Scale for Development [59,60] was used to evaluate Psychomotor development level. For each scale, the raw scores were converted to age-corrected standard scores with a mean of 100 and standard deviation of 15.

A General Quotient (GQ) and six sub-quotients were obtained. Locomotor sub-scale assesses gross motor skills, Personal-Social sub-scale measures proficiency in the activities of daily living and interaction with other children, Language sub-scale measures expressive and receptive language, Eye and Hand Co-ordination focuses on fine motor skills, manual dexterity and visual monitoring skills, Performance sub-scale assess non-verbal reasoning, Practical Reasoning assesses the ability to solve practical problems, understand basic maths concepts and moral issues.

A total of 71 subjects (26 NF1, 26 NF1 + OPG and 19 NF1 + CT) had Total, Verbal and Performance IQ/DQ.

### 4.3. Psychopathological Assessment

The presence of behavioral problems was assessed by psychopathological questionnaires Child Behavior Checklist, in the two versions 6–18 years and 1y½-5, respectively for school age and pre-school age children [61,62]. The questionnaires were administered to parents of 25 NF1, 24 NF1 + OPG and 16 NF1 + CT.

The norm-referenced CBCL is completed by parents and caregivers, and it describes a child’s functioning during the previous six months. The items measure specific emotional and behavioral problems on a three point Likert scale (0 = “Not True,” 1 = “Somewhat or Sometimes True,” or 2 = “Very True or Often True”). The technical manual reports good to excellent psychometric properties [61,63].

The CBCL contains two empirically-derived global scales and eight syndrome scales. The CBCL/6-18 version global Internalizing contains three syndrome scales: Anxious/Depressed, Withdrawn/Depressed, and Somatic Complaints. The global Externalizing Domain contains the Rule Breaking Behavior and Aggressive Behavior syndrome scales. Three other syndrome scales do not belong to either broadband scale: Social Problems, Thought Problems, and Attention Problems. A Total Problems scale quantifies overall impairment and is derived from the raw score sum of all eight syndrome scales. Raw scores for each scale are converted to norm-referenced T-scores (M = 50, SD = 10).

The CBCL/1½-5 version, the global Internalizing scale contains four syndromic scales (Emotionally reactive, Anxious/depressed, Somatic complaints and Withdrawn). Externalizing domain contains the Attention problems and Aggressive behavior scales. One other syndromic scale does not belong to any either Internalizing or Externalizing scale: Sleep problems. A Total Problems scale quantifies overall impairment and is derived from the raw score sum of all eight syndrome scales. Raw scores for each scale are converted to norm-referenced z-scores [63] and then to T-scores (M = 50, SD = 10).

For both the pre-school and school age versions, CBCL Diagnostic and Statistical Manual of mental disorders (DSM)-Oriented Scales are also present as a supplement the CBCL syndromic scales: Affective problems, Anxiety problems, Attention Deficit and Hyperactivity Disorder (ADHD) problems, Oppositional Defiant problems are common to 6–18y and 1y½–5y versions, Somatic problems and Conduct problems scales are present only in the school age CBCL and Pervasive Developmental problems scale is present only for pre-school CBCL.

“Pathological” scores are indicated by T-scores ≥64 on the global scales, and ≥70 on the syndromic and DSM-Oriented scales. “Borderline” ranges are considered from 60–63 and 65–69 on the global and syndromic scales, respectively.

### 4.4. Statistical Analysis

Statistical analyses were employed by SPSS Statistics 20 software (IBM Corporation, Armonk, NY, USA) [64]. One-way ANOVA was used to compare the standard scores on Developmental/Intelligence scales and CBCL scores for the three groups (NF1, NF1 + OPG, NF1 + CT), and post-hoc Bonferroni test was carried out to investigate between-group differences. Moreover, the observed frequencies of pathological CBCL scores were calculated for each scale with respect to cut-offs defined according to the Achenbach method, separated χ2-test and Fisher Exact test (when applicable) was used to compare the observed frequencies of patients’ pathological scores with those predicted for the normal population, and the adjusted Pearson residual analysis was performed to identify observed frequencies significantly higher or lower than expected frequencies.

## 5. Conclusions

In our knowledge, this is the first study comparing the influence of different type of brain lesions on emotional and behavioral outcomes in NF1 children. Despite preliminary, the present study adds insight on emotional and behavioral characterization of children with NF1 in cases where the disease is associated with the onset of tumors. In particular, present results confirm that the presence of brain tumors is related to more evident cognitive impairment, validating the visuo-spatial, attentive and visuo-motor domains as particularly important in the cognitive development of NF1 children [65]. Moreover, children with NF1 and additional diagnosis of brain tumor show in addition higher levels of behavioral problems, with special reference to children with optical pathways glioma.

These data can be useful to the clinical managing of these patients, confirming the need to provide systematically and comprehensive cognitive and emotional assessments to children with NF1, in addition to medical evaluations, especially when they present involvement of the central nervous system.

These data need to be further replicated overcoming the present limitations, in order to better characterize cognitive and emotional-behavioral outcome of these children according to the type of tumor (both histology and localization), possibly including measures of environmental variables such as parental stress level and social support and investigating the role of genotypic variability, with the aim to deepen understand how defects in the NF1 gene and associated genes create the diversity of clinical neuropsychiatric symptomatology.

## Figures and Tables

**Table 1:** Group comparison at the Intelligence and Developmental test.

Cognitive Tests	NF1	NF1 + OPG	NF1wCT	Total Sample	One-Way ANOVA			
	Mean (SD)	Mean (SD)	Mean (SD)	Mean (SD)	F	*p*-Value *	Post-Hoc ^§^	*p*-Value
Full IQ/GQ	99 (11)	86 (20)	84 (20)	90 (18)	5.27	0.007	NF1 > OPG	0.027
							NF1 > CT	0.018
							OPG > CT	0.999
Verbal IQ/DQ	98 (11)	87 (22)	90 (19)	92 (18)	2.77	0.07	n.s.	
Performance IQ/DQ	101 (13)	89 (18)	88 (19)	93 (17)	4.798	0.011	NF1 > OPG	0.034
							NF1 > CT	0.027
							OPG > CT	0.999
Block Design	10.5 (2.7)	7.6 (2.6)	8.5 (3.1)	9.0 (3.0)	6.733	0.002	NF1 > OPG	0.002
							NF1 > CT	0.082
							OPG < CT	0.999
Digit Span	8.7 (1.8)	8.1 (3.0)	6.2 (2.2)	7.9 (2.5)	4.113	0.022	NF1 > OPG	0.999
							NF1 > CT	0.019
							OPG > CT	0.136
Symbol search	10.52 (2.8)	9.0 (2.9)	7.7 (3.3)	9.3 (3.0)	3.335	0.044	NF1 > OPG	0.473
							NF1 > CT	0.044
							OPG > CT	0.786

Legend: NF1: neurofibromatosis type 1 controls children; NF1 + OPG: neurofibromatosis type 1 children with optical pathways glioma; NF1 + CT: neurofibromatosis type 1 children with other central nervous system lesions; CBCL: Child Behavior Checklist. * *p*-values of 0.05 or lower are considered significant. ^§ ^Post-hoc Bonferroni correction. IQ: Intelligence Quotient; GQ: General Quotient; DQ: Developmental Quotient. n.s.: not significant.

**Table 2:** Psychopathological ranges distribution among the three groups.

CBCL Measures	Problems Levels	NF1 N (%)	NF1 + OPG N (%)	NF1 + CT N (%)	χ2	*p*-Value
CBCL Int	Normal	12 (48.0)	14 (58.3)	12 (75.0)	10.412	**0.034**
	Borderline	8 (32.0)	1 (4.2)	1 (6.2)		
	Pathological	5 (20.0)	9 (37.5)	3 (19.0)		
CBCL Ext	Normal	20 (80)	15 (65.2)	14 (87.5)	6.245	0.182
	Borderline	3 (12)	2 (8,3)	0 (0.0)		
	Pathological	2 (8)	7 (29,2)	2 (12.5)		
CBCL Tot	Normal	15 (60)	12 (50)	13 (81,3)	5.114	0.276
	Borderline	4 (16)	5 (20,8)	0 (0)		
	Pathological	6 (24)	7 (29,2)	3 (18,1)		

Legend. NF1: neurofibromatosis type 1 controls children; NF1 + OPG: neurofibromatosis type 1 children with optical pathways glioma; NF1 + CT: neurofibromatosis type 1 children with other central nervous system lesions; CBCL: Child Behavior Checklist; Int: Internalizing score; Ext: Externalizing score; Tot: Total score.

**Table 3:** Comparisons of the Lesion Groups at CBCL Total Scores with and without controlling for IQ.

CBCL (T Score)	NF1 Mean (SD)	NF1 + OPG Mean (SD)	NF1 + CT Mean (SD)	One-Way ANOVA				One-Way ANOVA Controlling for IQ		
				F	*p*-Value *	post-hoc tests ^§^ NF1 < OPG NF1 < CNS	post-hoc tests OPG > CNS	F	*p*-Value	post-hoc tests NF1 vs. OPG NF1 vs. CNS OPG > CNS
Int Total	55 (11)	59 (13)	54 (12)	0.582	0.562	n.s.	n.s.	0.783	0.508	n.s.
Ext Total	51 (10)	56 (11)	51 (9)	1.438	0.245	n.s.	n.s.	3.357	0.024	n.s.
Total	55 (11)	58 (12)	53 (12)	0.981	0.381	n.s.	n.s.	1.902	0.139	n.s.
Attention problems	59 (8)	62 (11)	57 (9)	1.690	0.193	n.s.	n.s.	7.727	<0.001	n.s.
Oppositional deviant problems	55 (6)	58 (8)	52 (7)	3.388	0.040	n.s.	0.035	3.339	0.025	n.s.

Legend. NF1: neurofibromatosis type 1 controls children; NF1 + OPG: neurofibromatosis type 1 children with optical pathways glioma; NF1 + CT: neurofibromatosis type 1 children with other central nervous system lesions; IQ = Intelligence Quotient; CBCL: Child Behavior Checklist. * *p*-values of 0.05 or lower are considered significant. ^§^ Post-hoc Bonferroni correction.

**Table 4:** Age and sex distribution among the three groups.

Age/Sex	NF1 (*n* = 26)	NF1 + OPG (*n* = 26)	NF1 + CT (*n* = 19)	Total sample (*n* = 71)	Statistical Tests *	
Mean age months (SD)	112 (41)	107 (53)	90.53 (47)	104 (48)	F = 1.217	*p* = 0.303
Female (%)	12 (46.2%)	15 (57.7%)	6 (31.6%)	33 (46.5%)	χ^2^ = 3.011	*p* = 0.222

Legend. NF1: neurofibromatosis type 1 controls children; NF1 + OPG: neurofibromatosis type 1 children with optical pathways glioma; NF1 + CT: neurofibromatosis type 1 children with other central nervous system lesions. * *p*-values of 0.05 or lower are considered significant.

**Table 5:** Clinical characteristics of patients with NF1 and extra-optical pathways brain tumors.

ID	Sex	Age (months)	Total IQ	Verbal IQ	Performance IQ	Tumor localization *	Neurofibromas	T2H	CBCL Int T (0 = normal, 1 = borderline, 2 = clinical)	CBCL Ext T (0 = normal, 1 = borderline, 2 = clinical)	CBCL Tot T (0 = normal, 1 = borderline, 2 = clinical)
01CT	M	76	110	101	117	Brainstem (medulla)		Tha, SubTha, Br, BG, Hip/Amy, Cer	58 (0)	51 (0)	50 (0)
02CT	F	97	89	92	88	**Thalamus left**, CC sx (splenium), stage 3a	Peripheral and spinal	SubTha, Hip/Amy, Cer, WM			
03CT	F	163	59	78	56	Brainstem (middle inferior cerebral peduncle right)		Hip/Amy, Cer	78 (2)	66 (2)	72 (2)
04CT	F	177	93	90	106	Hypothalamus	Peripheral	Tha, Br, BG, Cer, WM	75 (2)	69 (2)	75 (2)
05CT	M	81	83	98	87	Brainstem (lateral pons right)		Tha, BG, Br, Hip/Amy, Cer	57 (0)	56 (0)	58 (0)
06CT	M	185	43	51	45	**Third ventricle (midbrain)**; Hypothalamus Basal Ganglia	Peripheral and spinal	Cer, Hip/Amy	N.a.		
07CT	M	43	69	72	88	Brainstem (medulla)		Tha, SubTha, Br, Hipp/Amy, Cer	48 (0)	38 (0)	66 (2)
08CT	F	42	69	89	79	**Basal ganglia left** (anterior internal capsule, globus pallidus, putamen); chiasma, hypothalamus	Peripheral	Br, Hipp/Amy, Cer	43 (0)	49 (0)	45 (0)
09CT	M	145	66	80	80	Brainstem (medulla)		Ta, SubTha, BG, Hip/Amy, Cer	N.a.		
10CT	M	65	97	96	100	Brainstem (pons and medulla)		SubTha, BG, Br, Hip/Amy, Cer, Coll	47 (0)	48 (0)	47 (0)
11CT	F	43	77	85	88	**Hypothalamus**; medulla		Tha, BG, Br, Cer, Hipp/Amy	40 (0)	49 (0)	43 (0)
12CT	M	50	102	108	93	Brainstem (pons and medulla)		Tha, BG, Br, Cer, Hip/Amy, Coll	59 (0)	58 (0)	59 (0)
13CT	M	60,5	62	53	78	**Brainstem (pons)**; bilateral cerebellar hemispheres dx>sx		Tha, BG, Br, Hip/Amy, Cer	59 (0)	55 (0)	59 (0)
14CT	M	26,5	65	69	62	Brainstem (pons and medulla left)		SubTha, Tha, BG, Br, Hip/Amy, Cer, Coll, WM	43 (0)	52 (0)	44 (0)
15CT	M	95	99	105	93	Brainstem (pons and medulla)		SubTha, Tha, BG, Br, Hip/Amy, Cer, Coll, WM	65 (2)	58 (1)	63 (1)
16CT	M	80	107	114	104	Brainstem (medulla right)		SubTha, Tha, BG, Br, Hip/Amy, Cer	50 (0)	48 (0)	45 (0)
17CT	F	80	94	98	100	Brainstem (pons and medulla left)		Tha, BG, Br, Hip/Amy, Cer	43 (0)	41 (0)	38 (0)
18CT	M	121	113	118	113	Hypothalamus	Peripheral	BG, Br, Hip/Amy	61 (1)	48 (0)	53 (0)
19CT	M	90	105	116	92	Brainstem (lamina quadrigemina)		SubTha, Tha, BG, Br, Hip/Amy, Cer	41 (0)	33 (0)	34 (0)

* For patients with more than one tumor, the most affected structure is bolded. Legend: SubTha: Sub-thalamic nuclei, Tha: Thalami; BG: Basal Ganglia; Br: Brainstem; Hip/Amy: Hippocampus/Amygdala; Cer: Cerebellum hemispheres and/or vermis; Coll: Colliculus; WM: White Matter; T2H: T2-Hyperintensities.

**Table 6:** Clinical characteristics of patients with NF1 and Optical Pathways Glioma.

ID	Sex	Age (months)	Total IQ	Verbal IQ	Performance IQ	Tumor localization *	Neurofibromas	T2H	CBCL Int T (0 = normal, 1 = borderline, 2 = clinical)	CBCL Ext T (0 = normal, 1 = borderline, 2 = clinical)	CBCL Tot T (0 = normal, 1 = borderline, 2 = clinical)
01OPG	F	53	106	120	90	1c left		SubTha, BG, Hip/Amy, Cer	52 (0)	54 (0)	52 (0)
02OPG	F	112	107	100	102	2b left	Peripheral	BG, Br, Hip/Amy, Cer	70 (2)	59 (0)	63 (1)
03OPG	M	170	79	86	87	1a left	Spinal	BG, Br, Hip/Amy, Cer, CC	44 (0)	43 (0)	42 (0)
04OPG	F	102	99	108	88	1a left		SubTha, Br, WM	49 (0)	43 (0)	48 (0)
05OPG	F	75	102	100	100	2a	Peripheral	BG, Hip/Amy, Cer	52 (0)	34 (0)	52 (0)
06OPG	M	50	69	48	82	Chiasm and Hypothalamus			59 (0)	61 (1)	62 (1)
07OPG	M	136	80	69	97	Chiasm and Hypothalamus					
08OPG	M	77	102	100	108	2a H+		SubTha, BG, Hip/Amy, Cer	77 (2)	67 (2)	77 (2)
09OPG	F	191	65	66	71	2a H+	Peripheral and spinal	BG, Hip/Amy, Cer	56 (0)	49 (0)	52 (0)
10OPG	F	141	114	107	118	3a H+	Spinal	Tha, BG, Br, Hip/Amy	59 (0)	56 (0)	54 (0)
11OPG	M	149	88	81	97	1a left	Peripheral and spinal	Tha, BG, Br, Hip/Amy, Cer, Coll	70 (2)	51 (0)	62 (1)
12OPG	F	127	103	92	106	1a right		SubTha, Tha, BG, Br, Hip/Amy, Cer	50 (0)	62 (1)	58 (0)
13OPG	M	43	37	33	39	2b right H+		SubTha, Tha, BG, Br, Hip/Amy, Cer	50 (0)	64 (2)	52 (0)
14OPG	F	206	79	86	93	2b right	Spinal	WM	76 (2)	56 (0)	66 (2)
15OPG	M	31	69	65	87	**3b H+**; basal ganglia		SubTha, Tha, BG, Br, Hip/Amy, Cer, WM	47 (0)	57 (0)	54 (0)
16OPG	F	83	110	96	119	3b H+		Tha, BG, Br, Cer, WM	33 (0)	41 (0)	36 (0)
17OPG	F	198	46	58	58	1a		SubTha, Tha, Br, Hip/Amy, Cer, WM	67 (2)	68 (2)	70 (2)
18OPG	F	100	66	76	65	2b	Peripheral	BG, Br, Hip/Amy, Cer	78 (2)	67 (2)	75 (2)
19OPG	M	41	97	106	87	3a H+		SubTha, Tha, BG, Br, Hip/Amy, Cer	41 (0)	77 (2)	60 (1)
20OPG	F	182	85	90	87	1a left	Spinal		62 (1)	55 (0)	63 (1)
21OPG	F	97	98	103	93	2b		BG	71 (2)	59 (0)	68 (2)
22OPG	F	110	101	120	102	1a left		SubTha, Tha, BG, Br, Hip/Amy, Cer, Coll	39 (0)	34 (0)	32 (0)
23OPG	M	30,5	69	69	75	**1c**; fornix		SubTha, Tha, BG, Br, Hip/Amy, Cer, Fornix	66 (2)	66 (2)	74 (2)
24OPG	M	60	83	84	93	2b left H+		BG, Br, Hip/Amy, Cer	66 (2)	71 (2)	73 (2)
25OPG	M	81	91	96	90	3b H+		SubTha, BG, Br, Hip/Amy, WM			
26OPG	F	130	97	106	87	**2b H+**; lamina quadrigemina		SubTha, Tha, BG, BR, Hip/Amy, Cer	59 (0)	47 (0)	54 (0)

* Optical Pathways Glioma categorization in accordance to Dodge et al. 1958, revised [52] and T2H information where available for 24 patients. For patients with more than one tumor, the optical pathways are the most affected structures. Legend: H+, Hypothalamic involvement; SubTha: Sub-thalamic nuclei, Tha: Thalami; BG: Basal Ganglia; Br: Brainstem; Hip/Amy: Hippocampus/Amygdala; Cer: Cerebellum hemispheres and/or vermis; Coll: Colliculus; WM: White Matter; T2H: T2-Hyperintensities.

## Data Availability

The neuropsychological data used to support the findings of this study are available from the corresponding author upon request.
